# Nonpuerperal breast abscess due to *Prevotella bivia*

**DOI:** 10.1002/ccr3.2824

**Published:** 2020-05-05

**Authors:** Astrid Boucher, Delphine Quaranta, Stéphane Emonet, Jacques Serratrice, Matteo Coen

**Affiliations:** ^1^ Breast Center Department of Gynaecology and Obstetrics Geneva University Hospitals Geneva Switzerland; ^2^ Service of Infectious Diseases Department of Medicine Geneva University Hospitals Geneva Switzerland; ^3^ Service of Internal Medicine Department of Medicine Geneva University Hospitals Geneva Switzerland; ^4^ Unit of Development and Research in Medical Education (UDREM) Faculty of Medicine University of Geneva Geneva Switzerland

**Keywords:** anaerobes, beta‐lactams, cultures, *Staphylococcus aureus*

## Abstract

Contrary to puerperal abscess, nonpuerperal breast abscess is often caused by anaerobic bacteria; polymicrobial aerobic‐anaerobic infections are also frequent. Empiric first‐choice treatment with broad‐spectrum antibiotics should be considered.

## Graphical abstract

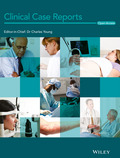

## 1 BACKGROUND

Most breast abscesses occur during lactation period as a rare, but serious complication of mastitis. Their incidence ranges from 0.4% to 11% of lactating women.^1^ They are due to milk stasis and bacterial proliferation. Bacteria of normal skin or oral flora are involved, especially *Staphylococcus aureus*.^2^, ^3^

Nonpuerperal (or nonlactational) breast abscesses are uncommon. Risk factors are trauma, obesity and smoking.^3^, ^4^, ^5^, ^6^ Other underlying conditions include rheumatoid arthritis or diabetes mellitus.^3^ Among the evoked pathogenetic mechanisms are periductal mastitis, infection arises in a cyst or a dilated duct,^7^ follicular obstruction of the pilosebaceous unit, or rarely granulomatous lobular mastitis.^8^ An inflammatory carcinoma is not excluded.^2^ Treatment regimens generally include antibiotics and drainage (either ultrasound‐guided or surgical).

This article describes the first case of nonpuerperal breast abscess due to *Prevotella bivia* in a 39‐year‐old woman. Antibiotic treatment for nonpuerperal breast abscess is discussed.

## 2 CASE REPORT

A 39‐year‐old woman with a history of IgA nephropathy (Berger's disease) and hypertension had consulted in the gynecological emergency unit complaining of a painful right breast mass that appeared 3 days earlier. She was gravida 6, para 5, and had breast‐fed all her children until the last one (one year before the consultation). She had no family history of breast cancer and no personal previous breast problems; she did not have diabetes and had never smoked.

The patient was afebrile. Physical examination revealed a 2‐cm tender breast mass under the right nipple, without any sign of local inflammation. Laboratory tests showed a normal total white cell count and mild elevation of C‐reactive protein (19 mg/dL). Breast ultrasound (US) revealed a 18 × 16 mm heterogeneous collection in the retro‐areolar area of the right breast; its hypervascularization of surrounding tissues was suggestive of abscess. An US‐guided aspiration yielded a purulent fluid that was sent for bacterial cultures. Empiric antibiotic therapy with oral flucloxacillin was started (500 mg/6 hours).

A follow‐up US performed 2 days after initiation of treatment showed progression of the collection (30 × 26 mm); a second aspiration was performed. Both samples of abscess fluid were positive for Gram‐negative bacilli (+++) and leukocytes (+++). Anaerobic culture was performed on CDC agar (bioMérieux SA) incubated at 37°C for 24‐48 hours in an anaerobic atmosphere (80% N_2_, 10% CO_2_, 10% H_2_) generated with the micro‐incubator M23C (Scholzen Microbiology Systems AG). Aerobic culture remained negative whereas anaerobic yielded a pure isolate of *Prevotella bivia*, identified by MALDI‐TOF/MS (Biotyper compass, Bruker Daltonics). At day 5, treatment was switched to oral amoxicillin‐clavulanate (625 mg/8 hours) and metronidazole (500 mg/8 hours), but on day 7, the mass had already further enlarged in size (50 × 30 mm) and became extremely painful. A new aspiration of the abscess was performed, but its culture remained “sterile”. Antibiotics were continued unchanged for 10 days; the mass eventually resorbed. Antibiotic susceptibility testing was performed using the ATB ANA® test (bioMérieux SA). The isolate was resistant to clindamycin but susceptible to amoxicillin‐clavulanate, piperacillin‐tazobactam, imipenem, and metronidazole. At 1‐month follow‐up, the patient was asymptomatic. Breast US and mammography showed neither residual collection nor recurrence; moreover, no underlying suspicious lesion was detected.

## 3 DISCUSSION

Most breast abscesses occur in young lactating women as a complication of mastitis or lactational breast inflammation. Milk stasis, leading to bacterial proliferation and tissue invasion, represents the pathogenetic key factor; bacteria of the resident skin flora, in particular *Staphylococcus aureus*, *Staphylococcus epidermidis,* and *Streptococcus* spp., are the prevalent causative agents.^1^, ^2^

Nonpuerperal breast abscesses are less frequent and classically associated with diabetes mellitus and smoking.^3^, ^4^, ^5^, ^6^ Other risk factors are trauma,^6^ obesity,^5^, ^6^ or rheumatoid arthritis.^9^ Interestingly, our patient lacked the risk factors usually associated with the disease (viz. she was a nondiabetic, nonsmoker woman in her reproductive years).

Among the evoked pathogenetic mechanisms are fistulas of lactiferous ducts by obstruction from to keratin plugs due to squamous metaplasia of lactiferous ducts epithelium (periductal mastitis or Zuska disease),^10^, ^11^, ^12^ infection arises in a stagnant fluid of a cyst or a dilated duct,^7^ or follicular obstruction of the pilosebaceous unit.^8^ Idiopathic granulomatous mastitis is an uncommon benign chronic inflammatory disease, which can clinically and radiographically mimic abscess or breast cancer. It is worth noticing that breast infections in the nonlactating woman should spur evaluation for an inflammatory carcinoma.^2^

Diagnosis is often clinical: A circumscribed and fluctuant breast mass, sometimes associated with erythema and orange‐peel (*peau d'orange*) skin, is the classic sign of a breast abscess; nonpuerperal abscesses are typically found under or around the areola, like in our patient, while puerperal abscesses are located more peripherally. Ultrasound is the preferred imaging method.

Abscesses smaller than 3 cm or puerperal abscesses are treated by needle aspiration, while larger abscesses normally require incision and catheter drainage.^13^ All patients are concurrently treated with empirical antibiotic therapy (vide infra, paragraph on microbiology and antibiotic treatment) and routinely revaluated at day 2. If the evolution is favorable, patients are seen again at day 14. If not, breast ultrasound and eventually needle aspiration may be repeated. Moreover, antibiotic therapy can be adapted based on microbiological results.

Breast imaging follow‐up at 3 months is offered to all patients in order to evaluate the mammary gland far from the infectious episode, and to rule of the presence of an underlying neoplasm.

Besides *Staphylococcus aureus*, cultures from nonpuerperal abscess often grow anaerobic bacteria like *Bacteroides* spp. and anaerobic Gram‐positive cocci (recovery rate 29.5%‐50%).^4^, ^13^, ^14^, ^15^, ^16^ Moreover, polymicrobial aerobic‐anaerobic infections are frequent.^17^

*Prevotella bivia* (previously *Bacteroides bivius*) belongs to the genus *Prevotella*, comprising anaerobic Gram‐negative bacteria typically isolated from the oral, upper respiratory, uro‐genital, and digestive tract^18^; Recently, *Prevotella* has been shown to be part of the normal microbiota of the human breast tissue.^19^ A few cases of nonpuerperal breast abscess caused by species of the genus *Prevotella* (*viz. Prevotella melaninogenica,* formerly *Bacteroides melaninogenicus*,^20^, ^21^, ^22^, ^23^ *Prevotella disiens*,^22^ *Prevotella intermedia*,^24^ *Prevotella timonensis* ^25^ and *Prevotella buccae* ^26^) have been described; nonpuerperal breast abscess due to *Prevotella bivia* have been mentioned^4^, ^13^ but have never been described until now.

Breast abscess, whether puerperal (lactational) or nonpuerperal, are often empirically treated with narrow‐spectrum (eg, nafcillin, oxacillin)—as we did in the case report before having the results of the bacteriological culture—and broader‐spectrum beta‐lactam antibiotics (eg, amoxicillin) active against Gram‐positive skin pathogens (*viz. Staphylococcus aureus*, *Staphylococcus epidermidis* and *Streptococcus* spp). However, this strategy does not take into account the microbiology of nonpuerperal breast abscess where anaerobes are among the primary pathogens (see before). Moreover, it is worth noticing that antibiotic resistance among anaerobia is increasing^27^; *Prevotella* spp. make no exception and decreasing penicillin susceptibility has been reported over the last years.^28^, ^29^, ^30^, ^31^

In case of nonpuerperal breast abscess, treatment with antimicrobials that are also active against anaerobes (eg, amoxicillin‐clavulanate) should be considered. If a treatment against Gram‐positive organisms is undertaken, broadening antimicrobial therapy to include anaerobes (eg, by adding metronidazole) must be granted in case of unfavorable evolution.

## CONFLICT OF INTEREST

None declared.

## AUTHOR CONTRIBUTION

AB: conceived or designed the work, and collected the data. DQ: conceived or designed the work. SE: involved in critical revision of the article and approved the final version of the manuscript to be published. JS: involved in critical revision of the article and approved the final version of the manuscript to be published. MC: conceived or designed the work, collected the date, drafted the article, involved in critical revision of the article, and approved the final version of the manuscript to be published.

## Data Availability

The data that support the findings of this study are available from the corresponding author, Matteo Coen, upon reasonable request.
